# News and Notes

**Published:** 1912-01

**Authors:** 


### NEWS AND NOTES.

### CIRCULAR I—ISSUED DECEMBER, 1911.

To Secretaries of Constituent Associations and of Component Societies and Councilors.

DEAR DOCTOR:—

This circular is issued to bear to you the compliments of the season, and for the purpose of establishing a helpful relationship between the Association, the constituent Associations, and their component societies. One of the most important offices of the national organization is to coordinate these component societies. With this end we view, we hope this circular may be acceptacle and serviceable. If its reception warrants, others will be issued from time to time to tell of the activities in the various societies and to serve as a medium for an exchange of ideas.

It will greatly assist this work if all societies send to this office programs and calls or announcements of all their meetings. If your society does not do this, kindly place on your mailing list "The American Medical Association, Secretary's Department, 535 Dearborn St., Chicago."

The publications of component societies that we now receive range from announcements of the time and place of meeting, which usually give the program of the session and in some instances publish the paperss, either in full or in abstracts, read at the preceding meetings; to bright, attractive little local medical "newspapers" which in addition to publishing the records of the meetings, keep the members of the society informed of happenings that interest the local profession. One county secretary writes a circular letter which is manifolded and in place of a printed call is sent to the members of his society. This saves the society the cost of printing.

One of these bulletins or calls notes several removals, a resignation, and announces the election of a new member, and in each instance a brief comment is made. Another calls attention to a number on the program for the next meeting, as follows:

Since the last meeting of our Society one of our members was called to attend a lady suffering from slight pain in the stomach, followed by vomiting of blood and passing of tarry stools. Within a few days the patient died. The doctor in attendance secured permission to make an examination of the stomach after death. He learned the cause of his patient's pain and saw the pathological condition that finally caused death. Would you be interested to know about this case? Certainly? A most valuable and never-to-be-forgotten lesson. Come to the October meeting and you shall hear all about this interesting condition.

In a third, the following comment is made:

In our last issue we published a list of the members of the Fayette County Medical Society, 97 in number. Below is a list of physicians in the county who are not members. Please look over this list and if you know of any additional names, send them to the secretary, who wishes to have the name of every physician in our county. Let us endeavor to reduce this list to the vanishing point. In its present condition, it is a reproach to our Society, and especially to the secretary.

Still another "boosts" itself and then calls for the support of it members:

For the last two years we have had the largest increase in membership of any society in the State. During the next two years we are going to ask the other County Societies to "watch us," for we are going to lead the way in all other lines.

At this time when the fall work of the organization is about to commence, it seems not inopportune that we should make certain resolutions within ourselves as to the part which we shall individually play in the life of the Society during the coming year. The Alleghany County Medical Society is our Society, and it will be what we make it. In every medical society there is a more or less limited group of members who regularly attend all meetings. A second group may be counted upon to attend meetings which are of peculiar interest to themselves. There is a third and larger group composed of men who seldom or never assemble with their fellow members. If we have been counted with the second or third group in the past, would it not be better to affiliate ourselves with the first group at once?

Here is a plan that might be of service in conveying Christmas cheer to your membership:

The Board of Councilors has also authorized the purchase of one bound copy of the A. M. A. book entitled "Nostrums and Quackery" for every member of the Los Angeles County Medical Association. Your name will be stamped on the cover in gold letters. These books are sold by the A. M. A. at $1.25 each. Read this book yourself, and then place it on your reception-room table. A copy of this book will be sent you direct from Chicago.

It would be of interest to know when the secretaries of the various county societies last saw or heard from the councilor of their district. Please let us know when you last consulted him and whether or not you have invited him to "drop in" on your society at one of its meetings. Of course, he is a welcome visitor, but his task will be more pleasant if he is assured that you want his presence. Have you reported to the councilor any good papers read by your members during the past year? If he knew of these, he might arrange to have them repeated at some neighboring societies. An exchange of courtesies between component societies that will provide for the presentation of the valuable contributions of members of one society to other societies will both assist in arranging interesting programs and stimulate men to prepare original papers; moreover, the papers will improve with each presentation, as the author will profit by the discussion which the paper brings forth. These discussions will broaden the author's grasp of his subject and teach him how best to present the theme. Of course, you will recommend the address, not the speaker. For unless you will stand sponsor for the paper, you should not ask the councilor to do so.

Councilors will be interested to know of a scheme used by one of their number in Texas, who, in calling a district meeting, used an outline map of the counties in his district. In the different counties, along with the name of the county, he entered the number of physicians in the county, and the number that belonged to its component society. On the envelope, he mimeographed a list of the counties, followed by three columns of figures: first, the number of physicians in the county; second, the number not members of the county society; and third, the number of members of the county organization. This unique presentation of the membership of the district must have stimulated the different societies to activity in increasing membership. We shall be happy to hear of any improvement on this plan, or anything that has proven effective in benefiting the organization.

To repeat: Kindly send a copy of all programs and announcements of meeting of your society to this office; let us know what problem you meet; when you have sovled them, tell how you did it. Let us have your help that the organization of the medical profession may accomplish its object in assisting every member to be more worthy of his place in its ranks. We trust it is unnecessary to assure you that this office is ready and anxious to cooperate with you in every way possible.

Wishing you personally a very pleasant Yuletide and hoping that you and the society you serve may have a very prosperous New Year, I am

ALEX R. CRAIG, Secretary.

---

### CLINICAL SOCIETY OF NEW YORK POLYCLINIC MEDICAL SCHOOL AND HOSPITAL—MEETING NOVEMBER 6TH, 1911.

Dr. Packard, presented a case of Caisson Disease simulating Locomotor Ataxia.

The patient, a man 55 years of age, came complaining of chronic rheumatism. His history was unique—was perfectly well until 10 years ago, when he began to work in the tunnel. This resulted in an attack of Caisson's Disease, with lightning pains in the right leg and thigh. There were no other symptoms and after recovery he returned to the Caisson and continued to work until a month ago. During his work in the tunnel he had four or five attacks.

Examination showed him to be suffering from a peculiar case of Caisson disease, which resembled Locomotor Ataxia. The symptoms were Argyll-Robertson pupil, Romberg's sign, lightning pains in right leg, some pain in left, and an osteo-arthritis of the knee.

The question arose as to whether he was suffering from Syphilis or Caisson Disease? Wassermann and Noguchi tests were negative, as were injection tests of spirochaeta pallida. He denied all history of Syphilis.

Dr. Beal said that in his opinion, not all cases of Locomotor Ataxia were due to Syphilis. This case had reacted negatively to Wassermann, Noguchi, and to the spirochaeta pallida test. From examination it looked like Tabes.

Dr. Gilday thought that 60% was low for specific Tabes cases. His own experience had shown a larger percentage. In regard to the value of the Wassermann or Noguchi reaction, preceding or in the stage of Tabes, he had had negative reactions despite a positive history of Syphilis. He felt that in post-spyhilitic cases, these reactions were not reliable.

Dr. Gilday inquired whether in post-syphilitic cases, the spirochaeta pallida had produced a degenration in the spinal cord, and if so, would the dea spirochaeto give a reaction?

Dr. Packard replied that the spirochaeta were not dead but dormant, and this condition was what made the injection of "606" effective.

Dr. Sinclair asked if the patient had been drinking any alcohol before the test were made, as it is well known that if a man drinks a glass or two of whiskey before the Wassermann of Noguchi tests are made, they are apt to give negative results. Even one glass of whiskey will effect the test.

Case for Differential Diagnosis between Cirrhosis of the Liver and Carcinoma.

Dr. Beal presented this case for diagnosis, because he wished to differentiate between enlargement of the spleen, in cirrhosis, and absence of the enlargement of the spleen in Carcinoma.

In his opinion, with Cirrhosis of the Liver there is always enlargement of the spleen, and with carcinoma never enlargement of the spleen. The patient shown was perfectly well up to May last, when he commenced to fail. In June he fell sick, and since then has lost half his weight. He has been ascitic, and been tapped once, and sixty to seventy ounces of fluid removed. The spleen did not seem particularly enlarged, but just over the pyloris, was a hard carcinomatous mass. The man had the carcinoma symptoms, but no pain.

Dr. Packard asked if there would not be bloody fluid in carcinoma? Dr. Beal replied that bloody fluid was not necessarily present. Dr. Packard said that in cirrhosis of the liver, blood tinged fluid was rare, unless it was due to the rupture of some small vessel made by the surgeon in entering the abdomen, and so causing a return flow which was blood tinged. In all cases It seemed to him, s'nce there was portal obstruction, cirrhosis because the portal circulation is obstructed. This case might be caused by a cirrhosis of the liver or carcinoma of the liver. of cirrhosis of the liver, there is enlargement of the spleen, might have large spleen. He believed that Carcinoma did have an enlarged spleen.

Dr. Strauss thought that care should be exercised in eliminating other diseases of the liver, which might simulate carcinoma and cirrhosis, in the case observed. Enlarged liver with nodules, has shown tubercular abscess on operation.

Dr. Gilday suggested that an exploratory incision might have solved the diagnosis, and it would do no more injury than tapping.

Dr. Tovey asked if Dr. Beal had made a rectal examination, as the Mayos have found metastasis of the rectum in carcinoma of the liver.

Dr. Reich said that malignancy was usually found with bloody fluid.

Dr. Beal said that in his experience the fluid in cirrhosis was of the same character as that in malignancy. He said he was glad that most of those present found no enlargement of the spleen, as he wished to bring out its normal size in carcinoma.

Two Cases Showing Results of Whitehead Operation for Hemorrhoids:

Presented by Dr Dryfus.

The first patient came complaining of severe itching and granulation, at the rectum. He had been operated on during the summer by a modification of the Ball operation, but was still complaining of several points of itching. He was in bed for a week or so, with loss of control, which subsequently had been restored.

The other case was operated upon in England six months ago, by the regular Whitehead method. This patient came under observation, complaining of itching, discharge, pain, with slight blood in the stools. Examination showed what looked like ulcer, but proved to be mucous membrane: too much having been cut off, it had not healed, and the man was continually irritated. The Ball modification of the Whitehead was very successful. Many of the supposed strictures of the rectum were due, after a regular Whitehead operation to the sloughing of the mucous membrane. In a primary union this symptom was obviated.

Dr. Lynch said that these two cases had been shown because the one operated upon the Whitehead method had been told that nothing could be done for him, while as a matter of fact, he was completely relieved by a simple plastic operation.

---

### CLINICAL SOCIETY OF NEW YORK POLYCLINIC MEDICAL SCHOOL AND HOSPITAL—MEETING

### DECEMBER 5TH, 1911.

Case of Tubercular Iritis.—Presented by Dr. E. S. Thompson.

The case was interesting as one of primary tubercular iritis. The patient was an Italion, 21 years of age, two years in this country. He has three brothers living and well and one sister who died at age of seven. No family history of Tuberculosis. He reacted positively to the Von Piquet test, but otherwise shows no evidence of Tuberculosis.

*Eye Symptoms.*—Besides the usual adhesions of the lens, he shows a well-marked grayish nodule, projecting from the periphery of the iris, which is rather characteristic. The three growths on the iris which it is most mportant to distinguish are Sarcoma, so-called Gumma, and Tubercular.

Sarcoma is usually a dark, distinct mark, which is characteristic. Gumma is usually on the margin, and is associated with a good deal of inflammation, but clears up very rapidly under mercury. Tubercle most frequently occurs at the limbus. In this case one can see a cheesy mass, extending from the limbus to the adjacent structures. With the Von Piquet react'on and the clinical appearance, it seems a fair clinical conclusion that it is Tubercle of the Iris.

These cases are generally secondary to some process of the lung, and the primary cases that have been reported are open to question. The prognosis in this case appears favorable and the patient will probably recover if properly treated.

In response to a query as to what treatment would be effective, Dr. Thompson said he had been impressed with the value of Tuberculin, and would start that treatment immediately. He would take the precaution however of having a Wassermann test made. Surgical treatment was not indicated.

Dr. Lynch said he supposed that the same principles would hold good in this case as in other types of T. B., viz.:—Good hygienic surroundings and good food.

Report of a Case of Cancer of the Stomach.—Presented by Dr. Kellog.

Dr. Kellog had oerated upon this case several days before and, although the patient was doing well in the ward, regretted that he could not show it.

The man was about 50 years of age. About three years ago, after business anxiety, he complained of pain in the stomach, during the earliest stages of digestion, and attacks of vomiting. Later the time of the pain changed: He was awakened with pain in the inght. After a time these symptoms disappeared.

Two months ago there was a recurrence of symptoms, he had some tenderness and pain, located in the gall bladder region. The pain occurred before he had entered the hospital, coming on four to four and a half hours after food. The vomitus was dark in color and foul smelling. He had no night pain during this attack. After reaching the hospital, the night pain came on and suggested duodenal location. Examination of stomach contents showed excessive Hcl., faint trase of Lactic acid, and a few Boas Bacilli.

The X-ray showed an interruption of the peristaltic wave at one point. Physical examination of the stomach showed no enlargement, and no tumor could be felt. From an examination of the stomach contents, and the X-ray findings, an exploratory incision was suggested. Operation disclosed a mass on the posterior portion of the stomach.

The interesting features of the case were the total absence of physical signs, and the establishment of the diagnosis by the X-ray findings. A gastro-enterostomy was performed successfully.

Dr. Lynch inquired what preparation of Bismuth was used.

Dr. Kellog said the ictures were taken by Dr. Busby, who uses a preparation of Bismuth in Buttermilk. He did not know whether subnitrate or subcarbonate was used.

Dr. Grant said he expected from the history of the case to find duodenal ulcer, the period which came so long after eating being suggestive. Examination of gastric contents he has not found of much value in cases of duodenal ulcer. The delayed period of pain is, however, of importance. It is claimed that increase of Hcl. is a characteristic of the condition. In several of his cases there was no increase, while in others there was decided diminution in Hcl.

Dr. Wightman said that simple ulcers of the stomach required care in the diagnosis and subsequent treatment, as a malignant condition frequently became engrafted upon simple ulcers and were prone to run a very acute course.

Dr. Yeomans emphasized the value of the X-ray pictures in carcinoma, as they were extremely helpful in locating the stie of the tumor. It facilitated finding the lesion on the operation table when time was valuable.

Dr. Edgerton said that the X-ray might be misleading, as in a case he had recently observed with Drs. Hays Nisbet and Foote. The X-ray plates showed interference with the peristaltic wave at the lower end of the greater curvature of the stomach, but operation proved a Meckel's diverticulum in the ileum near the cecum.

Dr. Lynch said that he did not think any particular method should be depended upon entirely, but that all should be taken together, not omitting the X-ray, or gastric content examination. It has been observed that subnitrate in quantity may have poisonous effect, for that reason Kirks and others have adopted the chloride of bismuth, which is harmless and suspends very easily in milk.

Dr. Lynch gives two ounces of Chloride of Bismuth suspended in kumyss at night, the bowels having been emptied by an enema, and the next morning two ounces more and thus combined, the X-ray picture gives both stomach and colon.

This may be done every three or four days for four or five pictures preceding operation until the desired picture is secured.

---

### CLINICAL SOCIETY OF NEW YORK POLYCLINIC MEDICAL SCHOOL AND HOSPITAL—MEETING OF JANUARY 8TH, 1011.

Strangulated Hernia Operated Upon Under Cocaine.—Case presented by Dr. Robert Brennan.

Patient was a man of 41 years of age and is single. His hernia was noticed at birth, and for sixten years he wore a truss. At sixteen he discontinued its use, but after four years became engaged in laborious work, and had to put it on again. One week ago ago the hernia came down, and in spite of his efforts, he could not replace it. Three days after it came down he began to vomit, and this continued for two days until he entered a hospital. He had a marked mitral lesion, which suggested the desirability of cocaine anaesthesia. On cutting down, the sac enclosing the hernia was liberated, letting out bloody fluid and a knuckle of black gut. Under hot applications to the gut, the color became restored in about an hour, and the wound was sewed up. His pulse promptly dropped from 140 to 100 after the operation, and has remained normal since.

Dr. Morrow said he had never operated upon a thyro-hernia, from the fact that discoloration of the gut is often due to traction upon the vessels, and that time might be saved by promptly relacing the gut in the abdominal cavity. He had demonstrated that normal gut, when constricted at the femoral ring, for a short period of time, would promptly lose its color and become black and would regain it when shoved back into the abdomen.

Thyro-Glossal Cyst. Case Presented by Dr. J. A. Bodine.

Dr. Bodine showed a specimen of Thyro-glossal Cyst, which he had removed from a case in its entirety. The case was somewhat uncommon. He had noticed from former careful observations with Dr. Myles, that these cysts had a way of returning after operation, or at least leaving a suppurating condition in the neck. To obtain success, the cyst must be dissected from the base of the tongue to the Thyroid gland, including the ructs. The cyst wall was thin and nothing should be left if success was to be obtainerd and a cure promised.

Dr. Morrow said that he had never oerated upon a thyroglossal cyst, but had a case of branchial cyst, which came under the class of congenital malformations. This case was very difficult because the wall of the fistula, which opened into the pharynx was adherent to the deep vessels, and it was very hard to separate them, but this was finally accomplished.

Amputation of Finger Tip with Replacement and Recovery of Finger.—Case Presented by Dr. J. A. Bodine.

Dr. Bodine showed a case of a little fellow who, while working at a cloth cutting device, in a clothing shop, caught his finger tip in the machinery, cutting off the finger, about half an inch from the tip. The boy was badly frightened, and had run to the hospital, leaving the finger tip on the floor in the shop. A friend was sent after it, and returned two hours later, bringing the tip, which was rather dried up, but softened after soaking in normal salt solution. The tip was put in place and bound up. The finger showed complete healing, and a round line of scar tissue where the junction had taken place.

Resection of the Posterior Root of the Tri-Facial Nerve.—Presented by Dr. J. A. Bodine.

Dr. Bodine showed a case of tri-facial neuralgia of long standing in a woman 60 years of age. She had tried injections of alcohol from time to time, but these finally failed to relieve her, and she was willing to have anything done. With the patient in a sitting position, Dr. Bodine had opened the skull in front of the ear, just in front of zygoma, the opening being 5 c. m. in diameter, the bone removed and the brain lifted up. The tri-facial ganglion was not removed, but he went behind it, and put a hook around the root and resected it. Twenty-four hours after the operation she was entirely free from pain, and is cured for the balance of her life. There was no particular difficulty about the operation although it was the first he had done of the kind, and so far as he knew was the first time it had been performed in this city. It was not removing the gangloin but the lifting out of the sensory root from the pons varolii which rendered the area very dangerous. It is not difficult, however, for anyone trained in surgery. He believed that he had separated the sensory from the motor roots, as mastication had not been interfered with. Such cases as had been treated by this method had been successful. At the beginning of the operation he had torn the middle meningeal artery, but there was no trouble checking the hemorrhage. Dr. Bodine thought that the fear of hemorrhage made cowards of many surgeons. The patient did not suffer from shock, and the pulse did not go above 90. The operation requires skill and judgment, as it is distinctly a one man operation from start to finish.

Dr. Keller had given this case of Dr. Bodine's four alcohol injections, with slight benefits. Alcohol injections usually give relief in these cases, and in his experience of fifty cases, he had had excellent results. Where relief was not obtained he thought that the lesion was further back than the injection reached. Even when a resection was contemplated, Dr. Keller thought it benefited the patient to precede the resection by alchol injection.

Dr. Connor welcomed any method of resection which spared the motor roots, as a gangloin resection usually resulted in a loss of the eye.

Case of Salpingitis Infection Following Conception.—Case Presented by Dr. Morgan.

Dr. Morgan showed a case of a woman 31 years of age, married, with one child 11 years of age, and a history of several miscarriages. Her husband stated that she was exposed to gonorrhea, but she gave no history of inconvenience. Four weeks before admission to hospital, she called her family physician, who found her in pain, and bleeding. She said she had missed one period, and was afraid she was going to miscarry. Bi-manual examination showed a mass posterior to the uterus, and a diagnosis was made of a probable ruptured Ectopic pregnancy. When seen a little later, there was a tremendous mass. On consultation she had been removed to the hospital and a laparotomy performed, and a pregnant uterus found. There was nothing to indicate a sub-mucous or an intra-mural fibroid. Further examination brought to light a tubo-ovarian cyst on the right side, and a chronic pyo-salpingitis of the left tube with the fimbriated extremity closed. The tube and ovary on the right side were removed, and the left tube also. Dr. Morgan was amazed that the woman was pregnant. She began to menstruate after a rest of two weeks in bed. The interesting feature was that the woman became pregnant after an infection which apparently closed both tubes.

---

### EXAMINATION OF DENTISTS FOR THE U. S. ARMY.

---

The Surgeon General of the Army announces that examinations for the appointment of Acting Dental Surgeons will be held at Fort Slocum, New York; Columbus Barracks, Ohio; Jefferson Barracks, Missouri; Fort Logan, Colorado; and Fort McDowell, California, on Monday, April 1, 1912.

Application blanks and full information concerning these examinations can be procured by addressing the "Surgeon General, U. S. Army, Washington, D. C."

The essential requirements to securing an invitation are that the applicant shall be a citizen of the United States, shall be between 21 and 27 years of age, a graduate of a dental school legally authorized to confer the degree of D. D. S., and shall be of good moral character and habits.

Acting Dental Surgeons are employed under a three years' contract, at the rate of $150.00 per month. They are entitled to traveling allowances in obeying their first orders, in changing stations, and in returning to their homes at termination of service. They also have the privilege of purchasing certain supplies at the Army commissiary. After three years' service, if found qualified, they are promoted to the grade of dental surgeon with the rank of first lieutenant, and receive thereafter the pay and allowances appertaining to that rank.

In order to perfect all necessary arrangements for the examination, applications must be in the possession of the Surgeon General at least two weeks before the date of examination. Early attention is therefore enjoined upon all intending applicants. There are at present 29 vacancies to be filled.

---

### U. S. CIVIL SERVICE EXAMINATIONS. COMPETITIVE EXAMINATIONS UNDER THE RULES OF THE U. S. SERVICE COMMISSION, FOR THE POSITIONS NAMED, WILL SOON BE HELD THROUGHOUT THE UNITED STATES.

### EXAMINATIONS TO BE HELD IN THE SPRING OF 1912.

*The following-named examinations will be held on various dates between March* 1 *and May* 1, 1912:

Apprentice plate printer, Bureau of Engraving and Printing.
Elevator conductor, departmental service.
Press feeder, Governmental Printing Office.
Stenographer, departmental, Philippine, and Isthmian Canal

Services.

Stenographer and typewriter, depratmental, Philippine, and Isthmian Canal Services.

Subclerical, departmental service.

Typewriter, departmental, Philippine, and Isthmian Canal Services.

*The following-named examinations will be held on March* 13, 1912.

Aid, Coast and Geodetic Survey.

Apprentice plate cleaner, transferrer, and engraver, Bureau of Engraving and Printing.

Assistant, Philippine Service.

Assistant examiner, Patient Office.

16—MEDICAL -------------- et aoinetaoin etaoinetaoin eta

Bookkeeper, Philippine Service.

Civil Engineer, Philippine Service.

Civil engineer student.

Computer, Nautical Almanac Office (men only).

Computer, Naval Observatory (men only).

Draftsman:

Copyist topographic.

Junior engineer, Engineer Department at Large.

Topographic, departmental.

Forest assistant, Forest Service.

Forest assistant, Philippine Service.

Industrial teacher (men only), Philippine Service.

Junior engineer (mechanical), Bureau of Mines.

Junior engineer (mining), Bureau of Mines.

Kindergarten teacher, Indian Service.

Local and assistant inspector of boilers.

Local and assistant insector of hulls.

Matron, Indian Service.

Physician.

Postal clerk, Isthmian Canal Service.

Teacher, Philippine Service.

Veterinarian, Philippine Service.

Veterinary inspector.

*The following-named examinations will be held on April 10, 1912:*

Agricultural inspector, Philipp ne Service.
Assistant observer, Weather Bureau.
Bookkeeper, departmental service.
Cadet engineer, Lighthouse Service.
Cadet officer, Lighthouse Service.
Civil engineer, departmental service.
Civil engineer and draftsman.
Computer, Coast and Geodetic Survey (men only).
Draftsman:
Mechanical, Isthmian Canal Service.
Topographic, Isthmian Canal Service.
Engineer, Indian Service.
Farmer, Indian Service.
Fish culturist.
Junior engineer (civil), Engineer Department at Large.
Junior engineer (mechanical or electrical), Engineer Department at Large.
Pharmacist, Public Health and Marine-Hospital Service.
Scientific assistant, Department of Agriculture.
Surveyor, Philippine Service.
Teacher, Indian Service.
Trained nurse.

*The railway mail clerk examination will be held on May* 4 1912.

The Commission has been unable to supply the demand for MALE stenographers and typewriters, especially at Washington, D. C. Young men who are willing to accept appointment at an entrance salary of $840 to $900 per annum have excellent opportunitiess of appointment. Advancement of capable appointees is reasonably rapid. The Governmental Service offers a desirable field to bright and ambitious young men.

In accordance with an act of Congress an applicant for examination in the apportioned Departmental Service at Washington, D. C., will be required to be examined in the State or Territory in which he resides, and to show in his application that he has been actually domiciled in such State or Territory for at least one year previous to the examination. This provision does not apply to other services.

Application forms and full information in regard to the above-named examination may be obtained by addressing the U. S. Civil Service Commission, Washington, D. C., or the Secretary of the Board of Examiners at the following-named places: Post office, Boston, Mass.; Philadelphia, Pa.; Atlanta, Ga.; Cincinnati, Ohio; Chicago, Ill.; St. Paul, Minn.; Seattle, Wash.; San Francisco, Cal.; Customhouse, New York, N. Y., New Orleans, La.; Old Customhouse, St. Louis, Mo.

JOHN C. BLACK, President.

---

### RED CROSS PRIZES.

The American Red Cross desires again to invite attention to the exhibition in connection with the Ninth International Red Cross Conference, which will be held in Washington, D. C., from May 7 to 17, 1912.

The exhibition will be divided into two sections, which will be styled Marie Feodorovna and General. The former is a prize competition, with prizes aggregating 18,000 rubles, or approximately $9,000, divided into nine prizes, one of 6,000 rubles, approximately $3,000; two of 3,000 rubles each, and six of 1,000 rubles each.

The subects for this competition are as follows:

1. A scheme for the removal of wounded from the battlefield with the minimum number of stretcher bearers.

2. Portable (surgeons') washstands, for use in the field.

3. The best method of packing dressings for use at first aid and dressing stations.

4. Wheeled stretchers.

5. Transport of stretchers on mule back.

6. aEsily folding portable stretchers.

7. Transport of the wounded between warships and hospital ships, and the coast.

8. The best model of portable Roentgen apparatus, permitting utilization of X-rays on the battlefield and at first aid stations.

The maximum prize will be awarded to the best exhibit, irrespective of the subject, and so on.

The General Exhibit is again divided into two parts; the first will be an exhibition by the various Red Cross Associations of the world. The second will be devoted to exhibits by individuals or business houses of any articles having to do with the amelioration of the sufferings of sick and wounded in war, which are not covered by the Marie Feodorovna Prize Competition for the year. While the American Red Cross will be glad to have any articles pertaining to medical and surgical practice in the field, especitlly anxious to secure a full exhibit relating to preventive measures in campaign. Such articles will be classed as follows:

1. Apparatus for furnishing good water in the field.

2. Field apparatus for the disposal of wastes.

3. Shelter such as portable huts, tents and the like, for hospital purposes:

4. Transport apparatus (to prevent the suffering of sick and wounded), exclusive of such apparatus as specified for the Marie Feodorvna Prize Competition.

As with the Marie Feodorovna Prize Competition, for this country only articles having the aproval of the Central Committee of the American Red Cross will be accepted.

Diplomas will we awarded for exhibits in this section of the exhibition as approved and recommended by the Jury.

Further information may be obtained from the Chairman, Exhibition Committee, American Red Cross, Washington, D. C.

It is perhaps to apparatus having to do with the prevention of disease in armies that the energies of Americans have been specially directed since the Spanish-American War. Therefore. the last mentioned section of the Exhibition should make an appeal to them.

